# Rescue of exotropia subsequent to pulled-in-two syndrome of the medial rectus muscle occurring during surgery for high myopic strabismus fixus: A case report

**DOI:** 10.1097/MD.0000000000031864

**Published:** 2022-12-30

**Authors:** Shinya Nakao, Manabu Miyata, Mariko Hirai, Shusaku Mizoguchi, Hirokazu Ohashi, Hirokazu Nishiwaki

**Affiliations:** a Department of Ophthalmology, Tenri Hospital, Tenri, Japan; b Department of Ophthalmology and Visual Sciences, Kyoto University Graduate School of Medicine, Kyoto, Japan.

**Keywords:** high myopia, medial rectus muscle, myopic strabismus fixus, pulled-in-two syndrome

## Abstract

Pulled-in-two syndrome is one of the significant complications of strabismus surgery. This study aimed to report a case of pulled-in-two syndrome of the contractured medial rectus muscle (MR) that occurred during strabismus surgery for strabismus fixus due to high myopia, and to describe a rescue of this complication.

**Patient concerns:**

A woman in her 60s presented to our Ophthalmology Department with the main complaint of unilateral high myopia and severe myopic strabismus fixus. Esotropia exceeded 45° and hypotropia exceeded 15° in her right eye in the Hirschberg test. Right eye duction was markedly limited in every gaze direction. Orbital magnetic resonance images showed rupture of the superior rectus to lateral rectus band ligament and lengthening of the distance between the SR and LR muscles in the right eye.

**Diagnosis:**

Due to the patient's ophthalmic examination and imaging results, she was diagnosed with high myopic strabismus fixus.

**Interventions:**

We performed MR recession and Yokoyama surgery to correct right eye hypoesotropia. In the MR recession procedure, pulled-in-two syndrome (MR muscle tear) occurred. Thus, no additional procedure was performed on the MR. After the surgery, she presented 45 prism diopter exotropia and 18 prism diopter residual right hypotropia in a Krimsky test. We performed a second surgery, combining MR muscle advancement and inferior rectus (IR) muscle recession, 3 months after the first surgery.

**Outcomes:**

One and a half years after the second surgery, she presented exotropia of 14 prism diopters without hypotropia in the Krimsky test and was satisfied with her ocular position and improved motility.

**Lessons:**

We experienced pulled-in-two syndrome in a case with severe myopic strabismus fixus and achieved a good outcome by performing additional surgery 3 months later, in which the lost MR muscle was advanced. This case underscores that, if the lost muscle cannot be found during surgery, one should maintain composure and perform a reoperation a few months after the initial surgery, if necessary. This case report can aid in making rescue treatment decisions when pulled-in-two syndrome occurs.

## 1. Introduction

Pulled-in-two syndrome is characterized by extraocular muscle rupture under strong tension during strabismus surgery. This is extremely rarely encountered in clinical practice, but is one of the significant complications of strabismus surgery.^[1]^ The clinical manifestations of pulled-in-two syndrome are related to limited duction in the field of action of the lost muscle. Major risk factors for pulled-in-two syndrome are cranial nerve palsy, prior surgery, and advanced age.^[2]^

We experienced a case of pulled-in-two syndrome of the contractured medial rectus (MR) muscle that occurred during strabismus surgery for treatment of severe myopic strabismus fixus. Furthermore, we rescued the complication 3 months after the surgery.

## 2. Case report

**Figure 1.**
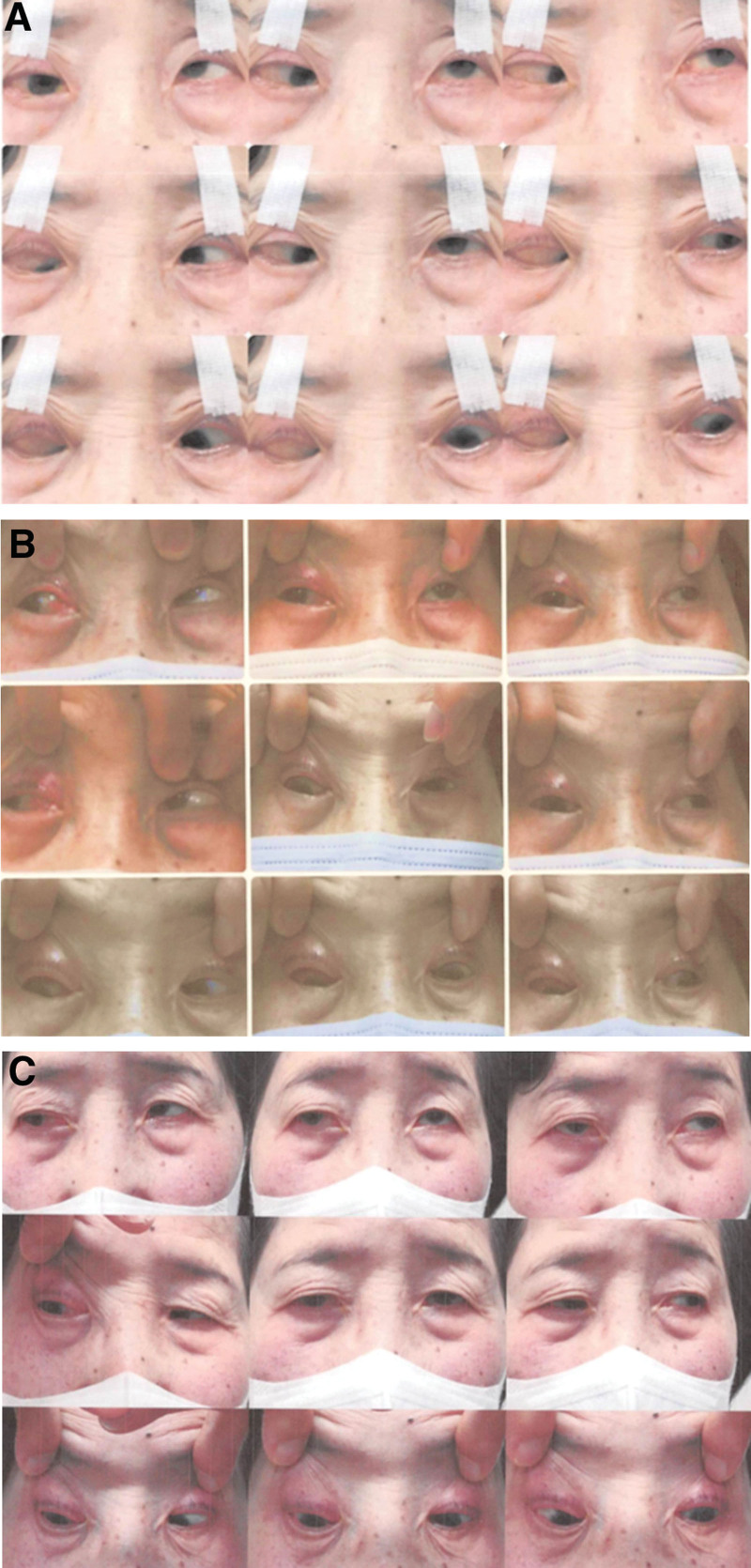
Photographs of 9 ocular gaze positions, before and after strabismus surgery. (A) Photographs before the first surgery. The patient presented hypoesotropia of the right eye in the primary gaze position. She had marked limitation of the supraduction and abduction of the right eye. (B) Photographs taken 3 months after the first surgery and before the second surgery. She presented hypoexotropia of the right eye in the primary gaze position. She had residual limitation of the supraduction while abduction limitation improved in the right eye. (C) Photographs 1.5 yrs after the second surgery. She presented a small angle of exodeviation without vertical deviation of the right eye in the primary gaze position. Ocular motility had improved markedly.

A 60s woman presented with unilateral high myopia and myopic strabismus fixus. She showed esotropia of more than 45° and hypotropia of more than 15° in the right eye in the Hirschberg test (Fig. 1A). She had marked limitation of duction of the right eye in every gaze direction, whereas duction of the left eye was normal. She had a medical history of Sjogren’s syndrome with dry eye, bilateral primary open-angle glaucoma, and hyperglobulinemia. She used latanoprost and a combination of dorzolamide hydrochloride and timolol maleate eye drops for both eyes and brimonidine tartrate eye drops for the left eye to treat glaucoma; and rebamipide eye drops to treat dry eye in both eyes. We had performed cataract surgery for the right eye 7 months before the strabismus surgery.

The best-corrected visual acuity was 20/1000 and 20/20 in the right and left eyes, respectively. The axial length was 26.23 mm and 22.98 mm in the right and left eyes, respectively. Color fundus photography revealed macular chorioretinal atrophy in the right eye. Magnetic resonance imaging of the orbit detected rupture of the superior rectus to lateral rectus band ligament, and enlargement of the distance between the SR and LR muscles in the right eye. These abnormal findings were absent in the left eye.

**Figure 2.**
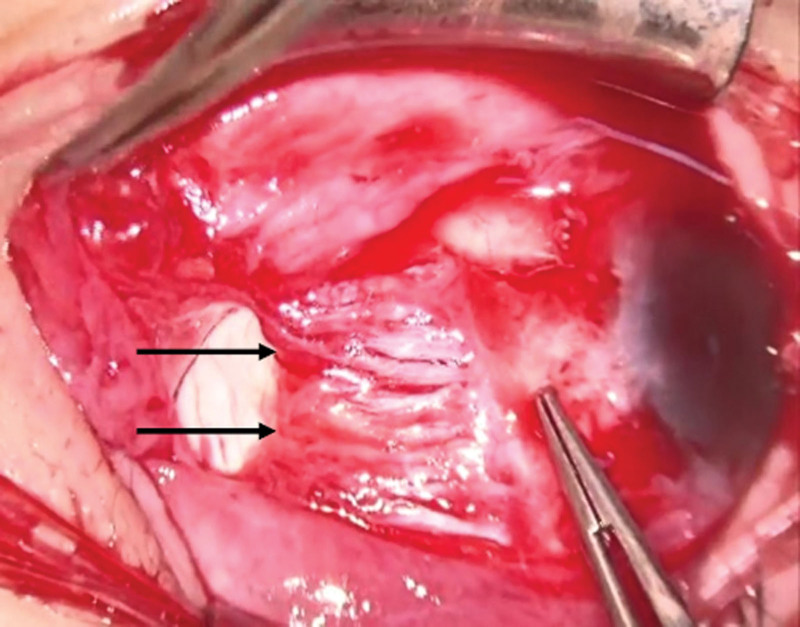
An intraoperative image of the medial rectus muscle in the first surgery. The medial rectus muscle was ruptured at the muscle belly approximately 5 mm from the insertion. Black arrows indicate the ruptured stump of the medial rectus muscle. Since this image was obtained from intraoperative movie, the image quality is poor.

We performed MR recession and Yokoyama surgery^[3]^ to correct the hypoesotropia of the right eye.^[4]^ An intraoperative forced duction test was positive in the abduction (the direction of stretch of the MR muscle) in the right eye. We performed a limbal conjunctival incision from the 8 o’clock to the 4 o’clock position and identified the LR, SR, and MR muscles. We cut Tenon’s capsule around the MR muscle, including the inter-muscle membrane and check ligament. Since the MR muscle was extremely difficult to pull because of the contracture, pulled-in-two syndrome (MR muscle tear) occurred during the MR recession procedure. The MR muscle ruptured at the muscle belly, approximately 5 mm from the insertion (Fig. 2). Although we extensively searched for the posterior-side of the MR muscle, we were unable to find it. We, therefore, performed no additional procedure on the MR muscle, and had to change the surgical procedure from recession to tenotomy of the MR muscle. We subsequently performed Yokoyama surgery as scheduled. We identified the LR and SR muscles and cut the Tenon’s capsule around the muscles. Then, we united the muscle bellies of the SR and LR muscles 15 mm posterior to the insertions using 3 knots of 5-0 polyester (Dacron, Alcon Japan, Tokyo, Japan). We sutured the conjunctiva using 8-0 silk (Alcon Japan).

**Figure 3.**
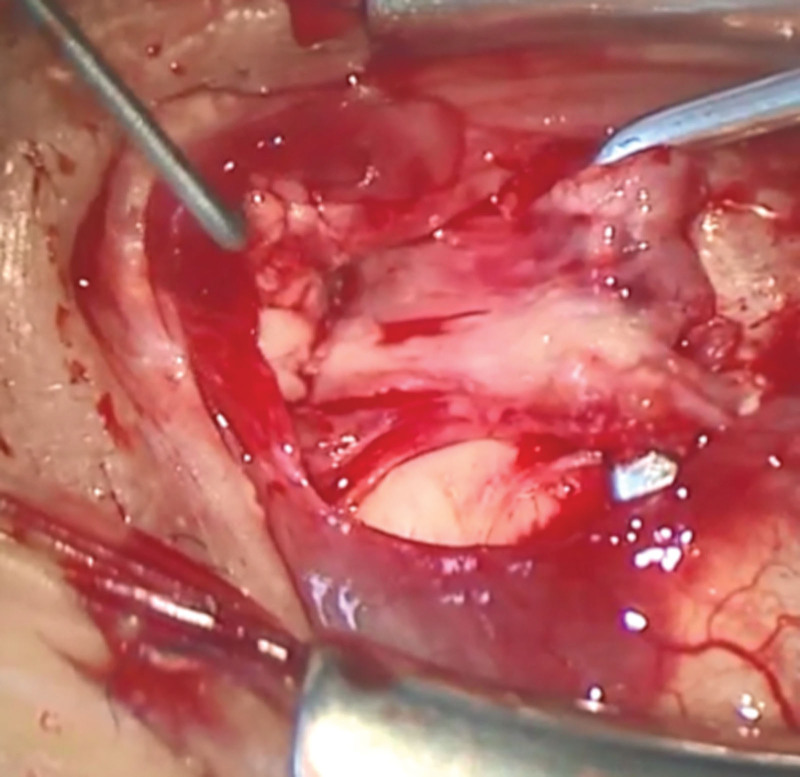
An intraoperative image of the medial rectus muscle in the second surgery. The pseudotendon of the medial rectus muscle was attached at the nasal sclera far from the insertion. The pseudotendon insertion was pulled by a strabismus hook. Since this image was obtained from intraoperative movie, the image quality is poor.

Three months after this surgery, she presented subsequent exotropia of 45 prism diopters and residual right hypotropia of 18 prism diopters in a Krimsky test (Fig. 1B). Therefore, we decided to perform a combination of MR muscle advancement and inferior rectus (IR) muscle recession 3 months after the first strabismus surgery. We performed a limbal conjunctival incision from the 2 o’clock to 7 o’clock position and identified the MR and IR muscles. We looked for the pseudotendon of the MR muscle, which would be attached at the nasal sclera, and easily found it distant from the original insertion (Fig. 3). We clamped the pseudotendon and true tendon of the MR muscle far enough from the insertion of the pseudotendon using strabismus forceps. Then, we performed advancement to the original insertion using 3 knots of 7-0 nylon (Ortho, Handaya, Tokyo, Japan). Furthermore, we performed IR recession of 5.0 mm in the usual manner.^[5]^ We sutured the conjunctiva using 8-0 silk.

One and a half years after the second strabismus surgery, the patient was satisfied with her ocular position. She presented exotropia of 14 prism diopters, without vertical deviation at near, in the Krimsky test. Furthermore, ocular motility had improved markedly (Fig. 1C).

## 3. Discussion

Contracture of the MR muscle occurs with long-standing myopic strabismus fixus. This causes marked limitation of abduction, with stretching of the MR muscle.^[6]^ Various surgical treatments are available for myopic strabismus fixus, including recession–resection surgery, Yokoyama’s surgery (uniting the muscle bellies of the SR and LR), and their combination.^[4,6]^ In our case, because the preoperative evaluation showed large-angle hypoesotropia of the right eye, we expected that recession–resection surgery would induce under-correction. Therefore, we decided to perform a combination of Yokoyama’s surgery and MR recession. In both recession–resection and in combination surgery, MR recession must be performed. Pulled-in-two syndrome occurred during MR recession in our patient. However, the lost MR muscle was easily detected in a subsequent second surgery, and MR advancement could be performed without difficulty, at 3 months after the first surgery. Thus, loss of the MR muscle during surgery should not cause undue stress.

In 1990, Greenwald and Parks described a rare complication of intraocular surgery, which occurred at the muscle-tendon junction, named “pulled-in-two syndrome,” an intraoperative tear of a rectus muscle caused by excessive tension or pull on the muscle or weakness in the muscle tissue.^[7]^ Pulled-in-two syndrome occurs in 0.14% of adults undergoing strabismus surgery.^[7]^ The complication occurs mainly in the MR or IR muscle, and particularly in the MR muscle (50%). The risk factors are advanced age, previous extraocular muscle surgery, extraocular muscle palsy, thyroid disease, the presence of a scleral buckle, and cranial nerve palsy.^[2]^ In our patient, the MR muscle had contracture with fibrosis due to the long-standing myopic strabismus fixus, thereby weakening the muscle. Additionally, strong tension was needed to stretch the muscle during the recession procedure. This case emphasizes the need for particular care during the high-risk MR recession in myopic strabismus fixus.

The best rescue of this complication is finding and reattaching the lost muscle to the globe during the surgery. However, the operator may decide not to reattach it, particularly in cases with large-angle strabismus. A previous study reported 2 cases of pulled-in-two syndrome occurring during strabismus surgery for myopic strabismus fixus, in which lost muscles could not be recovered, but an acceptable appearance was achieved; therefore, they required no additional treatment.^[8]^ However, our patient showed consequent exotropia of 45 prism diopters; therefore, MR muscle advancement was needed. Our experience highlights 2 crucial points: first, in myopic strabismus fixus, MR tenotomy might cause large overcorrection, and second, it is possible to find the pseudotendon with the slipped true tendon fixed to the sclera, even 3 months after the tenotomy. A previous case report recounted that a muscle lost intraoperatively was found 18 years after the first strabismus surgery.^[9]^ Since the necessity for additional surgery varies across individuals, decisions should be made based on postoperative evaluation after the first surgery.

## 4. Conclusion

Pulled-in-two syndrome is characterized by extraocular muscle rupture under strong tension. This intraoperative complication is rarely encountered in clinical practice, but its rescue is sometimes difficult during the surgery. The clinical manifestations of pulled-in-two syndrome are related to limited duction in the field of action of the lost muscle. Major risk factors for pulled-in-two syndrome are cranial nerve palsy, prior surgery, advanced age, and myopic strabismus fixus. Here, we reported our experience of a case of contracture-related pulled-in-two syndrome of the MR muscle that occurred during strabismus surgery for myopic strabismus fixus. Furthermore, we described a method for rescuing this complication when necessary after the initial surgery.

## Author contributions

**Conceptualization:** Shinya Nakao, Manabu Miyata.

**Project administration:** Manabu Miyata.

**Writing – original draft:** Shinya Nakao.

**Writing – review & editing:** Manabu Miyata, Mariko Hirai, Shusaku Mizoguchi, Hirokazu Ohashi, Hirokazu Nishiwaki.
